# Iron Supplementation Is Associated with Improvement of Motor Development, Hemoglobin Level, and Weight in Preterm Infants during the First Year of Life in China

**DOI:** 10.3390/nu14132624

**Published:** 2022-06-24

**Authors:** Suhua Xu, Liya Ma, Hailin Li, Xiaotong Wang, Miao Wu, Jiajia Jing, Xiaoyan Chen, Ruiling Lan, Weike Tang, Yanna Zhu

**Affiliations:** 1 Department of Maternal and Child Health, School of Public Health, Sun Yat-sen University, Guangzhou 510080, China; xush59@mail2.sysu.edu.cn (S.X.); lihlin29@mail2.sysu.edu.cn (H.L.); wangxt27@mail2.sysu.edu.cn (X.W.); wumiao6@mail2.sysu.edu.cn (M.W.); jingjj3@mail2.sysu.edu.cn (J.J.); chenxy727@mail2.sysu.edu.cn (X.C.); lanrling@mail2.sysu.edu.cn (R.L.); 15108252479@163.com (W.T.); 2 Shenzhen Bao’an Women and Children’s Hospital, Jinan University, Shenzhen 518102, China; maria226@sina.com; 3 Guangdong Provincial Key Laboratory of Food, Nutrition and Health, School of Public Health, Sun Yat-sen University, Guangzhou 510080, China

**Keywords:** iron supplementation, preterm, neurobehavioral development

## Abstract

Iron supplementation is recommended for preterm infants due to impaired iron endowment. However, the health outcomes of this recommendation remain controversial. Thus, this study aimed to determine the association of iron supplementation with neurobehavioral development, hemoglobin (Hb), and anthropometric characteristics in preterm infants. A retrospective cohort design was applied to collect data from 1568 preterm infants at 0–3 months of corrected age (mo CA) from a hospital in South China. Infants were categorized into a 3-month iron supplementation group (IG, *n* = 697) or a control group (CG, *n* = 871) according to medical records, and then followed through to 12 mo CA. Data on neurobehavioral development, anthropometry, Hb level, history of diseases, and nutrition were collected at 3, 6, and 12 mo CA. The results showed that, compared with the CG, iron supplementation was positively related to improved gross motor skills and weight at 6 mo CA (*β* = 1.894, *β* = 5.322) and 12 mo CA (*β* = 4.019, *β* = 6.830) and fine motor skills at 12 mo CA (*β* = 1.980), after adjustment for confounding factors including illness, nutritional supplements, and diet. Iron supplementation was also related to elevated Hb levels and its increase at 3 mo CA (*β* = 2.196, *β* = 3.920) and 6 mo CA (*β* = 3.011, *β* = 7.259). In conclusion, iron supplementation for 3 months in Chinese preterm infants is positively associated with improved motor development, elevated Hb levels, and higher body weight during the first year of life.

## 1. Introduction

An estimated 15 million babies are born prematurely every year, and this number is rising [1]. Globally, children face death and disabilities due to complications of preterm birth [1]. Preterm infants are at high risk of iron deficiency (ID) owing to impaired iron endowment caused by deprivation of iron accumulation in late pregnancy and pregnancy complications [2,3]. Moreover, early onset of erythropoiesis and rapid catch-up growth after birth require additional iron, especially in immature neonates [4,5]. It is estimated that unlike full-term infants, between 25% and 85% of premature newborns are more vulnerable to developing iron deficiency during the first 6 months of life [6]. ID can affect most organs, physical growth, anemia, and more importantly, metabolic processes of brain development, as confirmed in animal experiments [4,7,8]. Therefore, iron should be supplied in adequate amounts for premature infants.

Although iron supplementation is recommended for preterm infants [9], evidence for the impact of iron on neurodevelopment and growth is controversial [10,11,12,13]. Previous studies found that iron intervention during early infancy enhanced cognition and behavior [12,14,15,16], which indicated the importance of iron supplementation in early infancy. However, all of these studies focused on term infants. Moreover, most researchers focused on the effect of iron supplementation on cognitive development of preterm infants in the long term [17,18]. For instance, Berglund et al., reported no effects on cognitive outcomes at both 3.5 years and 7 years in low-birth-weight (LBW) infants given iron supplementation 6 weeks postnatally [17,18]. Little is known about the neurobehavioral development of premature infants with iron supplementation during the first year of life [19]. Additionally, the effect of iron supplementation on physical growth was inconsistent in the reported studies [11]. Some studies found that iron intervention in anemic or malnourished children improved physical growth [20,21,22], while a systematic review reported that iron supplementation had no effect on growth in the preterm and LBW infants [11]. The results could be confounded by other factors such as co-occurring nutritional deficiencies and illness or dietary factors including introduction of iron-rich complementary foods [23,24]. Therefore, it is essential to investigate the impact of iron supplementation on neurobehavioral development and physical growth in preterm infants taking confounders into consideration. Furthermore, duration of iron intake is closely related to the effect of iron supplementation. The evidence showed that iron supplementation for ≥8 weeks was likely to improve hemoglobin (Hb) in premature and LBW infants [11]. There was little benefit of short-term supplementation (<8 weeks) in this population [25,26].

Therefore, the objective of this study was to evaluate the association of iron supplementation in the first 3 months with subsequent neurobehavioral development in infants born prematurely (<37 weeks gestation), with confounding factors taken into account. The associations between iron supplementation in preterm infants and other health outcomes including Hb and physical growth were also investigated.

## 2. Materials and Methods

### 2.1. Study Design

The present study was a retrospective cohort study conducted in the outpatient section for high-risk infants in the Shenzhen Bao’an Women and Children’s Hospital, Jinan University, Shenzhen, China, from 2018 to 2020. Demographics and clinical information of newborn infants and data on neurobehavioral development, anthropometry, Hb level, and nutrition among infants at 3, 6, and 12 months of corrected age (mo CA) were collected from the medical records. The study was conducted in accordance with the Declaration of Helsinki, and the protocol was approved by the Ethics Committee of the School of Public Health Sun Yat-Sen University (project identification code: No. 2018005, date of approval: 3 February 2018).

### 2.2. Participants

Infants were eligible if they were premature (<37 weeks gestation), corrected age of 0–3 months after discharge, did not have obvious neurologic disorders, and were followed up at least once within 12 months of age. Preterm infants with serious progressive physical diseases, congenital disabilities, or congenital metabolic diseases were excluded during enrollment.

A total of 1568 preterm infants with 0–3 mo CA were enrolled in the study and were categorized as either the 3-month enteral iron supplementation group or the control group, according to medical records of iron supplementation throughout the child’s first 0–3 mo CA. The mean duration of oral iron supplementation was 2.94 ± 0.57 months. The mean dosage of iron supplementation was 1.76 ± 1.09 mg/kg per day based on the feeding guidelines for premature infants and the individual situation of premature infants [27].

### 2.3. Assessment of Outcomes

The primary outcome in infants was neurobehavioral development within 1 year postnatal. The Developmental Screening Test (DST) was used to evaluate the developmental status of infants at 3 mo CA with only a developmental quotient (DQ). The China Developmental Scale for Children (CDSC) was used to evaluate the neurobehavioral development of infants at 6 and 12 mo CA. The CDSC assessment comprises five specific domains: adaptability, gross motor skills, fine motor skills, language, and personal–social development. The DQs of the total domains and each domain were calculated for each participant as the ratio between the developmental age and corrected chronologic age, where a higher score means lower risk of developmental delay. Both the DST and the CDSC are suitable for the Chinese population and widely used in clinical practice in China due to high reliability and validity [28,29,30]. Trained staff conducted the DST and CDSC assessments in a specialized room with a quiet environment to ensure the infants’ emotional stability.

The secondary outcomes included Hb level and its increase from 1 mo CA, and anthropometry indices including length, weight, and head circumference. Anthropometric data were obtained by experienced clinicians and nurses. Anthropometry measurements were plotted on WHO growth charts to obtain a length-for-age Z score (LAZ); weight-for-age Z score (WAZ), and head circumference–for-age Z score (WAZ).

The trained staff were not aware of the infants’ iron supplementation status when they did the specific assessment.

### 2.4. Assessment of Covariates

Data on the basic characteristics of infants were collected from medical records only at recruitment and included demographic information, risk factors in the prenatal stage, and neonatal diseases. Data on infant nutrition were collected at follow up.

### 2.5. Demographics

Demographic information of newborn infants included sex, birth gestational age (GA), birth weight (BW), birth length (BL), and head circumference (HC). Preterm infants were divided into early preterm (<34 weeks) and late preterm (34–37 weeks). Infants were assigned to one of three BW groups: normal BW (NBW, BW ≥ 2500 g), low BW (LBW, 1500 g ≤ BW < 2500 g), and very low BW (VLBW, BW < 1500 g). BW was adjusted for GA and divided into three categories: small for gestational age (SGA), appropriate for gestational age (AGA), and large for gestational age (LGA).

### 2.6. Risk Factors in the Prenatal Stage

Prenatal risk factors consisted of parity, mode of delivery, singleton or not, and other diseases such as premature rupture of membrane, placenta previa, hypertensive disorders of pregnancy, gestational diabetes mellitus (GDM), and gestational iron deficiency anemia (IDA).

### 2.7. Risk Factors in the Neonatal Stage

Neonatal diseases related to subsequent neurobehavioral development comprised asphyxia at birth, neonatal intracranial hemorrhage, acute respiratory distress syndrome, neonatal hyperbilirubinemia, neonatal hypoglycemia, bronchopulmonary dysplasia, sepsis, infection, pneumonia, and neonatal anemia.

### 2.8. Nutrition during Follow Up

Data on infant nutrition were acquired from medical records based on face-to-face interviews using structured questionnaires with primary caregivers by a trained physician, which included feeding patterns (e.g., breast feeding or formula and mixed feeding), nutritional supplements, and complementary foods. Primary caregivers were asked about the dose of nutritional supplements including iron, vitamin A (vitA), vitamin D (vitD), and docosahexaenoic acid (DHA) at 3, 6, and 12 mo CA, as well as introduction of iron-rich foods (IRFs) such as fortified iron rice flour, animal liver or blood, and egg yolk at 6 and 12 mo CA.

### 2.9. Statistical Analysis

Data are presented as mean ± standard deviation (SD) or number (%). Normality was assessed using the Kolmogorov–Smirnov test. The *t* test or chi-square test was used to compare the differences in basic characteristics of infants at baseline, neurobehavioral development, Hb, and anthropometry at each visit between groups, when appropriate. A propensity score was estimated using a logistic regression model to avoid selection bias [31]. Covariates that were included in the propensity score model included risk factors in the prenatal and neonatal stage related to growth and development. Multiple linear regression models were performed to evaluate the association of iron supplementation with neurobehavioral development, Hb, change of Hb from 1 mo CA, and anthropometry at different ages, which is beneficial to observing the confounding factors at different stages, including iron-rich complementary foods and iron supplements at 6 and 12 mo CA. General estimating equation (GEE) models were also used to evaluate the association of iron supplementation with Hb and anthropometry under the whole period of follow up. Model 1 was unadjusted for confounders. Model 2 was adjusted for confounders including basic characteristics (e.g., infant sex and GA, BW, BL, or HC) and propensity score of risk factors. Based on model 2, nutritional information (e.g., feeding patterns, vitA, vitD, DHA, IRF) and CA at follow up were included as covariables in model 3. All statistical analyses were performed using R 4.0 (R Foundation for Statistical Computing, Vienna, Austria); *p* < 0.05 was considered statistically significant.

## 3. Results

### 3.1. Basic Characteristics of the Study Population

The flow chart of the participants is shown in Figure S1. No significant difference in adverse outcomes (death and illness) was observed between the iron supplementation group and the control group. Detailed information is shown in Table S1. The clinical characteristics of all 1568 participants treated with or without iron supplementation are reported in Table 1. Statistically significant differences in some variables were observed between the iron supplement infants and control group. The GA, BW, BL, and HC in the iron supplementation group were significantly lower than that in the control group (*p* < 0.001). A significantly higher prevalence of infants born early preterm (45.6%), LBW (68.9%), and VLBW (14.9%) was observed in the iron supplementation group than in the control group (*p* < 0.001).

The prevalence of neonatal disease in the iron supplementation group was more frequent than in the control group including neonatal intracranial hemorrhage (7.9% vs. 4.2%), acute respiratory distress syndrome (12.3% vs. 4.6%), neonatal hypoglycemia (7.7% vs. 4.8%), bronchopulmonary dysplasia (4.7% vs. 2.1%), infection (15.5% vs. 8.5%), sepsis (7.0% vs. 3.4%), pneumonia (25.1% vs. 15.2%), and neonatal anemia (34.1% vs. 18.8%). The iron supplementation group had a higher prevalence of GDM (22.5% vs. 18.9%) and gestational IDA (42.5% vs. 38.0%) than the control group (*p* < 0.05). There was no significant difference in sex, GA by weight at birth (AGA, SGA, LGA), and certain risk factors during pregnancy (parity, number of fetuses, premature rupture of membrane, and hypertensive disorders of pregnancy).

### 3.2. Differences in Neurobehavioral Development between the Iron Supplementation Group and the Control Group

Differences in infant neurobehavioral development between the iron supplementation group and control group 1 year postnatally are summarized in Table 2. The DQ at 3 mo CA in the iron supplementation group was 62.74 ± 12.98, significantly lower than in the control group (67.32 ± 13.34, *p* < 0.001). No difference in the overall DQ and DQ in each domain at 6 and 12 mo CA was observed between the control group and iron supplementation group (*p* > 0.05).

### 3.3. Differences in Hb and Anthropometry between the Iron Supplementation Group and Control Group

The level of Hb in the iron supplementation group (104.08 ± 11.78 g/L) was different from that in the control group (107.01 ± 11.49 g/L) at 1 mo CA (*p* = 0.008). The level of Hb was higher in the iron supplementation group at 3, 6, and 12 mo CA, with a statistically significant difference found only at 6 mo CA (*p* < 0.05). The mean increase of Hb at 3, and 6 mo CA from that at 1 mo CA was significant higher in the iron supplementation group than in the control group (*p* < 0.05).

There were statistically differences in LAZ, WAZ, and HAZ at 3 mo CA between the iron supplementation group and control group (*p* < 0.05). The LAZ and HAZ at 6 mo CA in the iron supplementation group were lower compared to the control group (*p* < 0.05). No difference in WAZ at 6 mo CA and LAZ, WAZ, and HAZ at 12 mo CA between the two groups was observed. Hb and anthropometric characteristics of infants between the iron supplementation group and control group are shown in Table 2.

### 3.4. Association of Iron Supplementation with Neurobehavioral Development

The association between iron supplementation and neurobehavioral development is shown in Table 3. Iron supplementation was negatively associated with total DQ at 3 mo CA (*β* = −4.577, 95% *CI*: −6.166, −2.988, *p* < 0.01) compared to the control group. However, no relationship between iron supplementation and total DQ at 3 mo CA was found after correction for sex, GA, and propensity score for risk factors in model 2 and further adjustment of feeding mode, nutrient supplements (e.g., vitA, vitD, DHA), and CA at follow up in model 3 (*p* > 0.05).

No significant association was observed between iron supplementation and total and subscale DQ at 6 mo CA in the crude model. Iron supplementation was positively related to total DQ (*β* = 1.669, 95% CI: 0.637, 2.701) and gross motor skills (*β* = 1.894, 95% CI: 0.344, 3.445), while no relationship was found in the other four functional areas after adjusting for sex, GA, propensity score for risk factors, feeding mode, nutrient supplements (e.g., vitA, vitD, DHA, iron), IRF, and CA at follow up (*p* > 0.05).

There was no significant relationship between iron supplementation and total DQ and subscale DQ at 12 mo CA in the crude model (*p* > 0.05). A significantly positive association was found between iron supplementation and total DQ (*β* = 1.759, 95% CI: 0.694, 2.824), gross motor skills (*β* = 4.019, 95% CI: 1.859, 6.178), and fine motor skills (*β* = 1.980, 95% CI: 0.471, 3.490) after adjustment for confounders. No significant association was detected between iron supplementation and adaptability, language, and personal–social skill at 12 mo CA regardless of adjustment for confounding factors.

### 3.5. Association of Iron Supplementation with Hb and Anthropometry

A significant negatively association of iron supplementation with Hb level was observed at 1 mo CA after confounding factors (*β* = −2.443, 95%CI: −4.356, −0.529, *p* < 0.05). The association of iron supplementation with Hb level and anthropometry in infants at 3, 6, and 12 mo CA is shown in Table 3. Compared to the control group, iron supplementation was significantly related to Hb levels at 3 mo CA (*β* = 2.196, 95% CI: 0.799, 3.594) and Hb increase from 1 mo CA (*β* = 3.920, 95% CI: 1.629, 6.211). Iron supplementation was positively associated with Hb level (*β* = 3.011, 95% CI: 1.395, 4.628) and its increase (*β* = 7.259, 95% CI: 3.939, 10.579) at 6 mo CA with adjustment for confounding factors. There was no significant association between iron supplementation and Hb levels and increase in Hb at 12 mo CA regardless of adjustment for confounding factors.

In comparison with the control group, iron supplementation was positively associated with WAZ at 6 mo CA (*β* = 5.322, 95% CI: 2.020, 8.625) and WAZ at 12 mo CA (*β* = 6.830, 95% CI: 2.730, 10.929) after adjusting for confounding factors such as sex, BW, propensity score for risk factors, feeding mode, nutrient supplements (e.g., vitA, vitD, DHA, iron), IRF, and CA at follow up. There was no significant association between iron supplementation and physical growth at 3 mo CA or LAZ and HAZ at both 6 and 12 mo CA after adjusting for confounding factors.

Additionally, GEE models were adopted to evaluate the relationship between iron supplementation and Hb and anthropometry. Compared to the control group, iron supplementation was positively related to Hb (*β* = 2.032, 95% CI: 0.808, 3.256), and weight (*β* = 4.267, 95% CI: 1.257, 7.270) during the first year of life (shown in Table S2).

## 4. Discussion

There is limited evidence to support the effects of iron on neurobehavioral development in preterm infants, and the impact of iron on growth remains inconsistent. The main purpose of this study was to evaluate the association of early iron supplementation with neurobehavioral development; secondary endpoints included Hb levels, Hb increase, and anthropometry indices in premature infants. The results obtained in the present study showed that, compared with no iron supplementation, early iron supplementation was positively associated with infants’ improved gross motor skills at 6 and 12 mo CA and fine motor skills at 12 mo CA, independent of the antenatal and neonatal risk factors, nutritional supplements, and dietary intake. Additionally, our study showed that early iron supplementation elevated Hb levels and its increase at 3 and 6 mo CA, as well as higher weight at 6 and 12 mo CA.

In the present study, we found that iron supplementation was associated with improved infant motor skills at both 6 and 12 mo CA, which is in accordance with previous findings in a study conducted on term infants receiving iron supplementation in the first 6 months [14,15,32,33]. Prior studies measuring motor outcomes in infants and toddlers found that iron-supplemented infants achieved higher motor scores at 6–12 months of age than infants administered placebo, but the difference was no longer significant at 18 months [14,15,16]. However, our findings are inconsistent with other studies [17,19,34,35]. For example, infants receiving iron supplementation from 6 months of age for 6 months showed no effect on motor skills at 12 months in comparison with the control group [19,35]. Furthermore, Friel et al., conducted a randomized trial of supplementation at two levels of iron intake for 1 year and measured motor development in LBW infants. Their study found no significant difference between treatment groups in assessment score at 3, 6, 9, or 12 mo CA [19]. Berglund et al., reported a lower prevalence of behavioral problems but no effects on cognition outcomes at both 3.5 years and 7 years in LBW infants where iron supplementation was initiated 6 weeks postnatally, compared with the placebo group [17,18]. This discrepancy may be partially due to the reference group, the initiation and duration of iron intake, or the timing of the assessment.

The scales used to assess later outcomes must be appropriately specific and not so global that subtle differences will not to be detected. Our finding showed that iron supplementation was associated with improved infant gross motor skills instead of fine motor skills at 6 mo CA and improved gross and fine motor skills at 12 mo CA in premature infants. However, the studies mentioned above did not distinguish between gross motor and fine motor skills when motor function was evaluated. Additionally, no association was found between iron supplementation in early infancy with language, adaptive, and personal–social development in preterm infants, which is consistent with previous studies [16,19]. Moffatt et al., and Friel et al., assessed the impact of iron supplementation on cognitive function and found no impact of iron supplementation on cognitive function or social–emotional development [16,19,36]. A plausible explanation for this is the corresponding development of specific brain regions and brain processes during the periods of iron supplementation. First, brain areas mature at different rates, and each area and process has specific iron requirements [36]. Different motor domains (e.g., reflexes, motor activity, postural control, sensory integration, and motor coordination) are subserved by various brain regions and networks. Previous studies have demonstrated that the complicated brain regions and pathways involved in motor development mature extremely rapidly in the first year of life, thus requiring more iron than other regions [37]. Moreover, iron is essentially required for myelin formation and oligodendrocyte function [2]. Therefore, brain pathways such as the corticostriatal and corticospinal tracts that are involved in obtaining motor skill may be susceptible to the effects of inadequate iron during infancy because these pathways are not comprehensively myelinated at birth [38]. Finally, motor performance changes with age. At 6 mo, infants are expected to exhibit trunk control (sitting), whereas infants are expected to exhibit more sophisticated trunk and extremity control at 12 mo, such as standing and buttoning a shirt [33].

Iron is an essential micronutrient, playing a critical role in Hb synthesis. This study found that, in comparison with the control group, iron supplementation throughout the first 3 months was correlated with improved Hb levels and its increase at 3 mo CA and 6 mo CA rather than 12 mo CA, which is consistent with previous studies. Prior RCT studies and follow up of RCT studies found that, if supplemented to 6 months of life, term infants may have improved Hb at 6 mo, with the effect not observed at 9 and 12 mo [14,19,34]. Thus, even if supplements are discontinued, the effect of iron on Hb can continue for a period but is not permanent. The relationship seems plausible because, as infants continue to grow and expand blood volume, iron is transferred from store and taken from supplements or the diet to blood compartment, making infants sufficient with regard to iron, which could be used to produce Hb [39]. Another potential explanation is that lower iron stores and higher iron requirements in infants born prematurely may downregulate the expression of hepcidin, a key regulator of systemic iron metabolism, thereby facilitating iron absorption and circulating iron release for Hb synthesis in maturing erythroblasts [4].

In our study, we found that iron supplementation was positively related to WAZ at 6 mo CA and 12 mo CA but had no association with LAZ and HAZ. There was evidence that iron supplementation had no effect on body length and head circumference in infancy. Berglund et al., conducted an RCT and found that iron supplementation until 6 months protected marginally low-birth-weight infants from iron deficiency and did not affect anthropometric data during the first 12 months of life [34]. Lozoff, B. et al., assessed the effects of iron supplementation in infancy from 6 weeks to 9 months and/or pregnancy on infant growth at 9 mo and found no group differences overall or among infants who were iron sufficient at birth [40]. In contrast, studies have shown an improved effect of iron supplementation on growth in malnourished or anemic children. A double-blind RCT showed that children with anemia aged 17–19 months who received iron for 2 months had an increased rate of weight gain [21]. Morais, M.B. et al., demonstrated a significant increase in WAZ but not in HAZ after iron therapy [22]. This association did not increase height and head circumference but increased weight and was essentially beneficial to infant growth. One possible mechanism of the association may be that rapid catch-up growth occurred in infants born with LBW [41]. In our studies, we observed that the iron supplementation group had a lower BW and higher prevalence of LBW and VLBW than the control group. The iron supplementation group showed faster catch-up growth and gained weight and height more rapidly due to recovery from poor fetal growth. However, catch-up in height was less than that in weight [42]. Moreover, the positive association of iron supplementation and higher weight may be correlated with gut microbiota involved in metabolic activity of energy [42]. Iron supplements could improve ID-related gut microbiota disorders and the intestinal microenvironment, thus contributing to the absorption of iron and other nutrients as well as weight gain in infancy [43].

One of the several strengths in our study is that, in focusing on premature infants, we have clarified the relationship of iron supplementation and health outcomes including Hb, growth, and neurobehavioral development, which is beneficial to clinical practice. Additionally, confounding factors such as demographic information, neonatal diseases related to subsequent neurodevelopment, risk factors in the prenatal stage, nutrients supplements (e.g., vitD, vitA, and DHA), and diet (e.g., IRF) were all considered in our study.

There are some limitations in the present study. First, attrition is an important limitation in this study that should be acknowledged. The reason for a high attrition rate in the visits was that parents prefer to take their infants to primary care institutions, such as community health service centers, due to greater geographical accessibility and cheaper services than large women and children’s hospitals. Though the attrition rate was high, no significant difference of adverse outcome (death and illness) for attrition was observed between the control group and the iron supplement group. Second, our study only considered the risk factors during pregnancy, including parity, GDM, and IDA, and did not add the social demographic characteristics of mothers. Third, data on nutrition parameters, baseline iron status or anemia before infants started iron supplementation and iron biomarkers at follow up were not collected, thus the association between iron administration and iron metabolism was not analyzed in this study.

## 5. Conclusions

For Chinese premature infants, iron supplementation for the first 3 months was positively associated with elevated Hb levels during the first 6 months and higher weight and improved motor development during the second half-year of life, especially in gross and fine motor skills, indicating that iron supplementation in early infancy is beneficial to not only iron status but also growth and neurobehavioral development in Chinese preterm infants during the first year of life. The pro-oxidative properties of iron also give rise to the need for extra caution and careful prescription by a physician performing frequent medical checks.

## Figures and Tables

**Table 1:** Demographic characteristics, neonatal diseases, and prenatal risk factors in the control group and iron supplementation group at birth.

Variables	Control Group	Iron Supplementation Group	*p*
	871	697	
**Demographics at birth**			
Sex			0.129
Boy	459 (52.7)	395 (56.7)	
Girl	412 (47.3)	302 (43.3)	
GA (weeks)	34.79 ± 2.06	33.59 ± 2.42	<0.001
GA group			<0.001
Late preterm	661 (75.9)	379 (54.4)	
Early preterm	210 (24.1)	318 (45.6)	
BW (g)	2271.47 ± 512.26	1997.13 ± 499.40	<0.001
BW group			<0.001
NBW	301 (34.6)	113 (16.2)	
LBW	502 (57.6)	480 (68.9)	
VLBW	68 (7.8)	104 (14.9)	
GA by weight at birth			0.635
AGA	746 (85.6)	596 (85.5)	
SGA	106 (12.2)	90 (12.9)	
LGA	19 (2.2)	11 (1.6)	
BL (cm)	45.87 ± 3.72	43.88 ± 4.14	<0.001
HC (cm)	31.70 ± 2.07	30.79 ± 2.28	<0.001
**Neonatal diseases**			
Asphyxia	38 (4.4)	38 (5.5)	0.379
Intracranial hemorrhage	37 (4.2)	55 (7.9)	0.003
Acute respiratory distress syndrome	40 (4.6)	86 (12.3)	<0.001
Hyperbilirubinemia	289 (33.2)	176 (25.3)	0.001
Hypoglycemia	42 (4.8)	54 (7.7)	0.022
Infection	74 (8.5)	108 (15.5)	<0.001
Bronchopulmonary dysplasia	18 (2.1)	33 (4.7)	0.005
Sepsis	30 (3.4)	49 (7.0)	0.002
Pneumonia	132 (15.2)	175 (25.1)	<0.001
Neonatal anemia	164 (18.8)	238 (34.1)	<0.001
**Prenatal risk factors**			
Parity			0.715
Nulliparous	497 (57.1)	405 (58.1)	
Multiparous	374 (42.9)	292 (41.9)	
Mode of delivery			0.011
Cesarean	499 (57.3)	380 (54.5)	
Natural labor	340 (39.0)	306 (43.9)	
Others	32 (3.7)	11 (1.6)	
Type of gestation			0.699
Singleton	688 (79.0)	557 (79.9)	
Multiple	183 (21.0)	140 (20.1)	
Premature rupture of membrane	280 (32.1)	232 (33.3)	0.672
Placenta previa	149 (17.1)	64 (9.2)	<0.001
Hypertensive disorders of pregnancy	63 (7.2)	53 (7.6)	0.856
GDM	165 (18.9)	157 (22.5)	0.093
Gestational IDA	331 (38.0)	296 (42.5)	0.082
**Propensity score**	0.41 (0.13)	0.49 (0.15)	<0.001

Gestational age, GA; birth weight, BW; normal birth weight, NBW; low-birth-weight, LBW; very low-birth-weight, VLBW; appropriate for gestational age, AGA; small for gestational age, SGA; large for gestational age, LGA; birth length, BL; head circumference, HC; iron deficiency anemia, IDA; gestational diabetes mellitus, GDM. Data are expressed as mean ± SD, or *n*(%). *t* test and chi-square test performed when appropriate. Significant differences, *p* < 0.05.

**Table 2:** Difference in neurobehavioral development, Hb, and anthropometry between the control group and iron supplementation group at 3, 6, and 12 months.

Variables	Control Group	Iron Supplementation Group	*p*
	871	697	
3 mo CA			
Neurobehavioral development			
DQ	67.32 ± 13.34	62.74 ± 12.98	<0.001
Hb, g/L	110.46 ± 11.74	111.68 ± 10.60	0.136
Change of Hb, g/L	5.46 ± 9.94	8.86 ± 10.84	0.013
Anthropometry			
LAZ	34.80 ± 28.48	22.77 ± 24.96	<0.001
WAZ	40.88 ± 29.51	34.90 ± 28.79	0.001
HAZ	29.00 ± 25.36	21.73 ± 23.67	<0.001
6 mo CA			
Neurobehavioral development			
DQ	84.99 ± 9.14	85.14 ± 8.56	0.839
Gross motor	85.33 ± 12.28	85.40 ± 11.62	0.944
Fine motor	85.60 ± 10.23	84.79 ± 11.17	0.352
Adaptability	85.84 ± 11.32	85.84 ± 11.80	0.992
Language	82.45 ± 11.45	82.54 ± 11.54	0.924
Personal–social	85.65 ± 11.70	85.27 ± 10.85	0.679
Hb, g/L	113.36 ± 11.93	116.37 ± 9.90	0.002
Change of Hb, g/L	6.95 ± 14.49	14.81 ± 11.33	<0.001
Anthropometry			
LAZ	40.64 ± 29.55	35.90 ± 28.55	0.017
WAZ	44.87 ± 30.18	45.70 ± 30.07	0.685
HAZ	38.29 ± 28.47	33.35 ± 26.99	0.009
12 mo CA			
Neurobehavioral development			
DQ	82.20 ± 7.86	82.04 ± 7.00	0.829
Gross motor	86.44 ± 14.38	87.13 ± 12.18	0.606
Fine motor	83.49 ± 9.83	83.68 ± 9.63	0.842
Adaptability	80.17 ± 10.19	79.10 ± 11.81	0.317
Language	78.72 ± 12.15	77.97 ± 11.34	0.523
Personal–social	81.38 ± 9.31	81.00 ± 9.19	0.678
Hb, g/L	120.23 ± 8.88	120.42 ± 7.90	0.847
Change of Hb, g/L	15.45 ± 12.93	17.44 ± 11.61	0.431
Anthropometry			
LAZ	41.39 ± 29.48	39.74 ± 28.81	0.504
WAZ	42.49 ± 28.76	45.72 ± 29.19	0.186
HAZ	43.55 ± 28.44	44.91 ± 30.20	0.582

Months of corrected age, mo CA; developmental quotient; DQ; hemoglobin, Hb; length-for-age Z score, LAZ; weight-for-age Z score, WAZ; head circumference–for-age Z score, HAZ. Data are expressed as mean ± SD. *t* test was performed. Significant differences, *p* < 0.05.

**Table 3:** The associations between iron supplementation with neurobehavioral development, Hb, and anthropometry in premature infants at 3, 6, and 12 months.

Variables	Model 1	Model 2	Model 3
3 mo CA			
Neurobehavioral development			
DQ	−4.577 *** (−6.166, −2.988)	−0.267 (−1.710, 1.176)	−0.136 (−1.607, 1.334)
Hb, g/L	1.218 (−0.123, 2.559)	1.112 (−0.320, 2.543)	2.196 *** (0.799, 3.594)
Change of Hb, g/L	3.402 ** (1.150, 5.654)	2.790 ** (0.469, 5.112)	3.920 *** (1.629, 6.211)
Anthropometry			
LAZ	−12.030 *** (−14.744, −9.317)	−3.722 ** (−6.256, −1.188)	−3.056 (−5.634, −0.479)
WAZ	−5.980 *** (−8.951, −3.009)	−5.980 *** (−8.951, −3.009)	2.431 (−0.288, 5.151)
HAZ	−7.263 *** (−9.794, −4.731)	−2.901 (−5.641, −0.162)	−2.096 (−4.865, 0.673)
6 mo CA			
Neurobehavioral development			
DQ	0.147 (−1.044, 1.338)	2.317 *** (1.283, 3.351)	1.669 *** (0.637, 2.701)
Gross motor	0.069 (−1.537, 1.676)	2.408 *** (0.904, 3.912)	1.894 ** (0.344, 3.445)
Fine motor	−0.807 (−2.233, 0.619)	1.404 (0.095, 2.714)	1.077 (−0.297, 2.451)
Adaptability	−0.009 (−1.553, 1.535)	2.278 *** (0.831, 3.725)	1.400 (−0.097, 2.897)
Language	0.090 (−1.449, 1.628)	2.297 *** (0.850, 3.744)	1.170 (−0.300, 2.640)
Personal–social	−0.382 (−1.901, 1.136)	1.474 (−0.012, 2.959)	1.232 (−0.309, 2.773)
Hb, g/L	3.011 *** (1.395, 4.628)	2.815 *** (1.128, 4.502)	2.752 *** (1.094, 4.409)
Change of Hb, g/L	7.860 *** (4.720, 11.000)	7.860 *** (4.573, 11.147)	7.259 *** (3.939, 10.579)
Anthropometry			
LAZ	−4.743 ** (−8.011, −1.475)	0.482 (−2.589, 3.552)	0.582 (−2.561, 3.725)
HAZ	0.837 (−2.550, 4.224)	5.094 *** (1.905, 8.284)	5.322 *** (2.020, 8.625)
WAZ	−4.944 *** (−8.066, −1.822)	−2.025 (−5.560, 1.511)	−1.249 (−4.867, 2.370)
12 mo CA			
Neurobehavioral development			
DQ	−0.161 (−1.382, 1.061)	1.851 *** (0.795, 2.907)	1.759 *** (0.694, 2.824)
Gross motor	0.690 (−1.508, 2.888)	3.241 ** (1.116, 5.366)	4.019 *** (1.859, 6.178)
Fine motor	0.192 (−1.388, 1.772)	2.198 ** (0.715, 3.681)	1.980 ** (0.471, 3.490)
Adaptability	−1.070 (−2.828, 0.688)	0.525 (−1.215, 2.265)	0.413 (−1.345, 2.171)
Language	−0.745 (−2.665, 1.174)	1.492 (−0.367, 3.350)	1.403 (−0.479, 3.284)
Personal–social	−0.379 (−1.879, 1.122)	1.403 (−0.035, 2.840)	0.825 (−0.596, 2.246)
Hb, g/L	0.189 (−1.423, 1.802)	−0.036 (−1.705, 1.633)	0.877 (−0.781, 2.535)
Change of Hb, g/L	1.984 (−2.148, 6.116)	0.763 (−3.646, 5.172)	2.652 (−1.864, 7.168)
Anthropometry			
LAZ	−1.647 (−5.696, 2.403)	3.093 (−0.804, 6.990)	2.582 (−1.642, 6.807)
HAZ	3.233 (−0.783, 7.248)	7.549 *** (3.765, 11.332)	6.830 *** (2.730, 10.929)
WAZ	1.357 (−2.696, 5.411)	3.160 (−1.471, 7.792)	3.480 (−1.366, 8.326)

Months of corrected age, mo CA; developmental quotient; DQ; hemoglobin, Hb; length-for-age Z score, LAZ; weight-for-age Z score, WAZ; head circumference–for-age Z score, HAZ. Data are generalized linear model coefficient (95% CI), refer to control group. ** *p* < 0.01; *** *p* < 0.001. Model 1, univariate analysis. Model 2, adjusted for sex, gestational age, and propensity score of 18 risk factors. Gestational age was adjusted when analyzing neurobehavioral development and Hb. Birth length, birth weight, and birth head circumference were adjusted when analyzing LAZ, WAZ, and HAZ, respectively. Model 3 adjusted for feeding patterns, nutritional supplements, and CA of follow up based on model 2. Iron-rich complementary foods and iron supplements were adjusted in model 3 only at 6 and 12 mo CA, respectively.

## Data Availability

The datasets used during the current study are available from the corresponding author on reasonable request.

## Supplementary Materials

The following supporting information can be downloaded at: https://www.mdpi.com/article/10.3390/nu14132624/s1, Table S1. Difference in nutritional information between the control group and iron supplementation group at 3, 6, and 12 months. Table S2. The associations between iron supplementation with Hb and anthropometry in premature infants during the first year of life. Figure S1. Flowchart of participants.

## Abbreviations

AGA, appropriate for gestational age; BL, birth length; BW, birth weight; CDSC, the China Developmental Scale for Children; DHA, docosahexaenoic acid; DQ, developmental quotient; DST, the Developmental Screening Test; GA, gestational age; GDM, gestational diabetes mellitus; HAZ, head circumference–for-age Z score; HC, head circumference; ID, iron deficiency; IDA, iron deficiency anemia; IRF, iron-rich food; LAZ, length-for-age Z score; LBW, low-birth-weight; VLBW, very low-birth-weight; LGA, large for gestational age; mo CA, months of corrected age; NBW, normal birth weight; PS, propensity score; RCT, randomized clinical trial; SGA, small for gestational age; vitA, vitamin A; vitD, vitamin D; WAZ, weight-for-age Z score.
