# Supplementary material for: Iron Supplementation Is Associated with Improvement of Motor Development, Hemoglobin Level, and Weight in Preterm Infants during the First Year of Life in China
Source: Nutrients. 2022 Jun 24;14(13):2624. doi: 10.3390/nu14132624 (PMC9267941; doi:10.3390/nu14132624)
Supplement: Supplementary file 1 [file nutrients-14-02624-s001.zip › nutrients-1724268-supplementary.pdf]

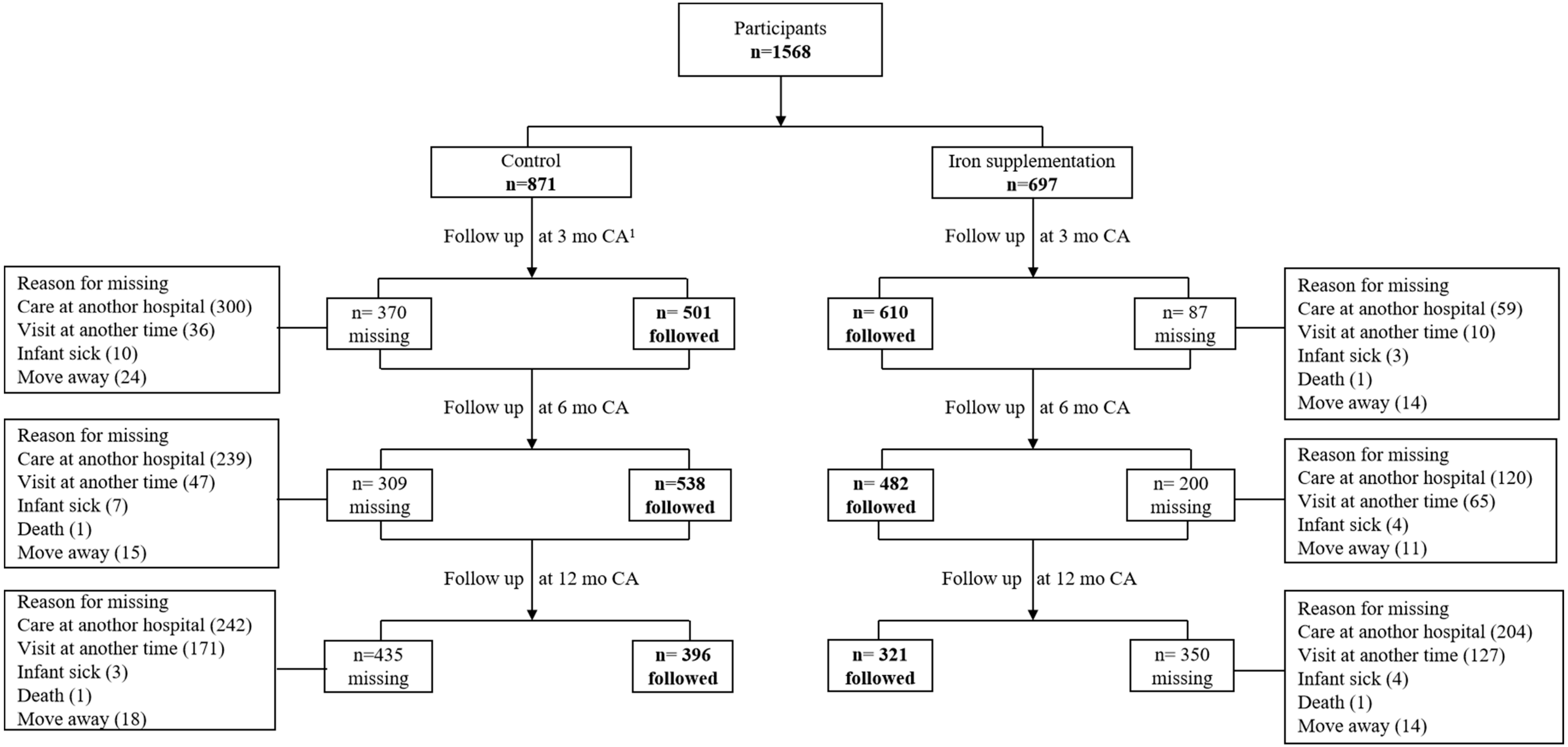

Figure S1 Flowchart of participants.

[1] Month of corrected age, mo CA.

Table S1 Comparison of adverse outcome (death and illness) between the control group and iron supplementation group at 3, 6, and 12 months

Variables	Control group	Iron supplementation group	*P*
3 mo CA	10(2.70)	4(4.60)	0.317
6 mo CA	8(2.59)	4(2.00)	0.772
12 mo CA	4(0.92)	5(1.42)	0.522

Data were expressed as n (%). Fisher tests were used to compare the proportion of death and illness of attrition reason.

Month of corrected age, mo CA

Table S2 The associations between iron supplementation with Hb and anthropometry in premature infants during the first year of life

Variables	Model 1	Model 2	Model 3
Hb	1.602(0.381,2.824)**	1.426(0.134,2.719)**	2.032(0.808,3.256)***
LAZ	-7.117(-10.137,-4.097)**	-0.708(-3.524,2.108)	-2.092(-5.319,1.135)
WAZ	-1.497(-4.758,1.764)	4.210(1.210,7.209)***	4.267(1.257,7.277)***
HAZ	-4.447(-7.391,-1.503)***	0.989(-1.792,3.77)	1.202(-1.564,3.968)

Hemoglobin, Hb; length-for-age Z score, LAZ; weight-for-age Z score, WAZ; head circumference -for-age Z score, HAZ;

Data are generalized estimating equations coefficient (95% CI), refer to control group. $^{**}p<0.05$; $^{***}p<0.01$.

Model 1, Univariate analysis;

Model 2, adjusted for sex, gestational age and propensity score of 18 risk factors. Gestational age was adjusted when analyzing Hb. Birth length, birth weight and birth head circumference was adjusted when analyzing LAZ, WAZ, HAZ, respectively.

Model 3, adjusted for feeding patterns, nutritional supplements, and CA of follow-up based on Model 2.
